# Temporal and anatomical distribution of ^18^F-flutemetamol uptake in canine brain using positron emission tomography

**DOI:** 10.1186/s12917-020-2240-y

**Published:** 2020-01-17

**Authors:** Taesik Yun, Wonguk Lee, Ji-Houn Kang, Mhan-Pyo Yang, Byeong-Teck Kang

**Affiliations:** 1 Veterinary Teaching Hospital, College of Veterinary Medicine, Chungbuk National University, Cheongju, Chungbuk 28644 South Korea; 2 Department of Nuclear Medicine, Chungbuk National University Hospital, Cheongju, Chungbuk 28644 South Korea

**Keywords:** Amyloid, Cognitive dysfunction syndrome, Dog, ^18^F-flutemetamol, Positron emission tomography

## Abstract

**Background:**

Positron emission tomography (PET) is increasingly being used as an imaging modality for clinical and research applications in veterinary medicine. Amyloid PET has become a useful tool for diagnosing Alzheimer’s disease (AD) in humans, by accurately identifying amyloid-beta (Aβ) plaques. Cognitive dysfunction syndrome in dogs shows cognitive and pathophysiologic characteristics similar to AD. Therefore, we assessed the physiologic characteristics of uptake of ^18^F-flutemetamol, an Aβ protein-binding PET tracer in clinical development, in normal dog brains, for distinguishing an abnormal state. Static and dynamic PET images of six adult healthy dogs were acquired after ^18^F-flutemetamol was administered intravenously at approximately 3.083 MBq/kg. For static images, PET data were acquired at 30, 60, and 90 min after injection. One week later, dynamic images were acquired for 120 min, from the time of tracer injection. PET data were reconstructed using an iterative technique, and corrections for attenuation and scatter were applied. Regions of interest were manually drawn over the frontal, parietal, temporal, occipital, anterior cingulate, posterior cingulate, and cerebellar cortices, cerebral white matter, midbrain, pons, and medulla oblongata. After calculating standardized uptake values with an established formula, standardized uptake value ratios (SUVRs) were obtained, using the cerebellar cortex as a reference region.

**Results:**

Among the six cerebral cortical regions, the cingulate cortices and frontal lobe showed the highest SUVRs. The lowest SUVR was observed in the occipital lobe. The average values of the cortical SUVRs were 1.25, 1.26, and 1.27 at 30, 60, and 90 min post-injection, respectively. Tracer uptake on dynamic scans was rapid, peaking within 4 min post-injection. After reaching this early maximum, cerebral cortical regions showed a curve with a steep descent, whereas cerebral white matter demonstrated a curve with a slow decline, resulting in a large gap between cerebral cortical regions and white matter.

**Conclusion:**

This study provides normal baseline data of ^18^F-flutemetamol PET that can facilitate an objective diagnosis of cognitive dysfunction syndrome in dogs in future.

## Background

Alzheimer’s disease (AD), the most common cause of dementia, is a degenerative brain disease [1, 2]. The typical symptoms of AD are difficulties with memory, language, and cognitive and other problem-solving skills, which affects a person’s ability to carry out normal activities [3]. AD is histopathologically characterized by accumulation of amyloid-beta (Aβ) plaques and neurofibrillary tangles [4]. Although a definitive diagnosis of AD depends on histopathological evaluation of brain tissue obtained at biopsy or autopsy [4], the recently approved in vivo amyloid positron emission tomography (PET) imaging provides an important new tool for increasing the objectivity of antemortem diagnosis of AD [5–9].

In vivo detection of amyloid deposition in the brain is now possible using ^11^C-based and ^18^F-labeled amyloid imaging agents in PET [5–12]. To date, six amyloid PET tracers have been commonly used: (a) ^11^C-Pittsburg compound B, (b) ^18^F-NAV4694 (AstraZeneca, Cambridge, England), (c) ^18^F-florbetapir (Amyvid, Avid Radiopharmaceuticals, Philadelphia, PA, USA. and Eli Lilly Co., Indianapolis, IN, USA), (d) ^18^F-florbetaben (Neuraceq, Piramal Imaging, Berlin, Germany), (e) ^18^F-flutemetamol (Vizamyl, GE Healthcare, Arlington Heights, IL, USA), and ^18^F-florapronol (Alzavue, FutureChem, Seoul, Korea). Among 6 amyloid PET tracers, ^18^F-flutemetamol PET has reported that the specificity and sensitivity of PET imaging evaluation in AD patients and older healthy control subjects was 85.3–93.3% and 93.1–97.2%, respectively [7, 10]. Visual evaluations of amyloid by PET using other ^18^F-based tracers have also shown that specificity ranging from of 91 to 100% and sensitivity ranging from 80 to 93% for discriminating AD in humans [11, 13].

Cognitive dysfunction syndrome (CDS) is a progressive neurodegenerative disease of geriatric dogs and cats. CDS shows cognitive and pathophysiologic changes that share many characteristics of AD, such as deposition of Aβ protein [14], of which the amino acid sequence is known to be identical between humans and dogs [15]. Diagnosis of CDS is subjective, because it is primarily based on recognition of cognitive and behavioral changes and exclusion of other medical diseases and adverse drug reactions that can mimic CDS [16].

Currently, there are few standardized diagnostic imaging criteria that can help to discriminate between dogs with and without CDS. Magnetic resonance imaging (MRI) is commonly applied in geriatric dogs. Typical MRI findings are: (1) ventricular enlargement [17], (2) widened sulci [17], and (3) decreased interthalamic adhesion thickness [18]. However, these findings show great variability, depending on breed and size [19–21], and it is difficult to apply these signs as criteria for the diagnosis of CDS.

Like human, Aβ deposition, in the form of diffuse senile plaques, is a widely reported characteristic of the aged canine brain [22]. It is also known that the amino acid sequence of Aβ protein is identical between humans and dogs [15]. Laboratory research on aged dogs have shown that the degree of Aβ deposition is strongly associated with impaired learning ability [23, 24]. Therefore, amyloid PET imaging may allow non-invasive in vivo measurements of Aβ plaques in the brains of living animals, and may promote understanding of amyloid accumulation across the CDS spectrum [25–29].

To our knowledge, there have been no reports on the use of ^18^F-flutemetamol PET in the veterinary literature. As with any imaging modality, it is important to know the physiologic state of ^18^F-flutemetamol uptake in normal canine brain to allow distinction of abnormal amyloid deposition. Therefore, the main purpose of this study was to assess the regional ^18^F-flutemetamol uptake in the normal canine brain over time, to facilitate use of ^18^F-flutemetamol PET in diagnosis of CDS in future.

## Results

### Positron emission tomography/computed tomography fusion images and SUVs in the normal canine brain

**Fig. 1:**
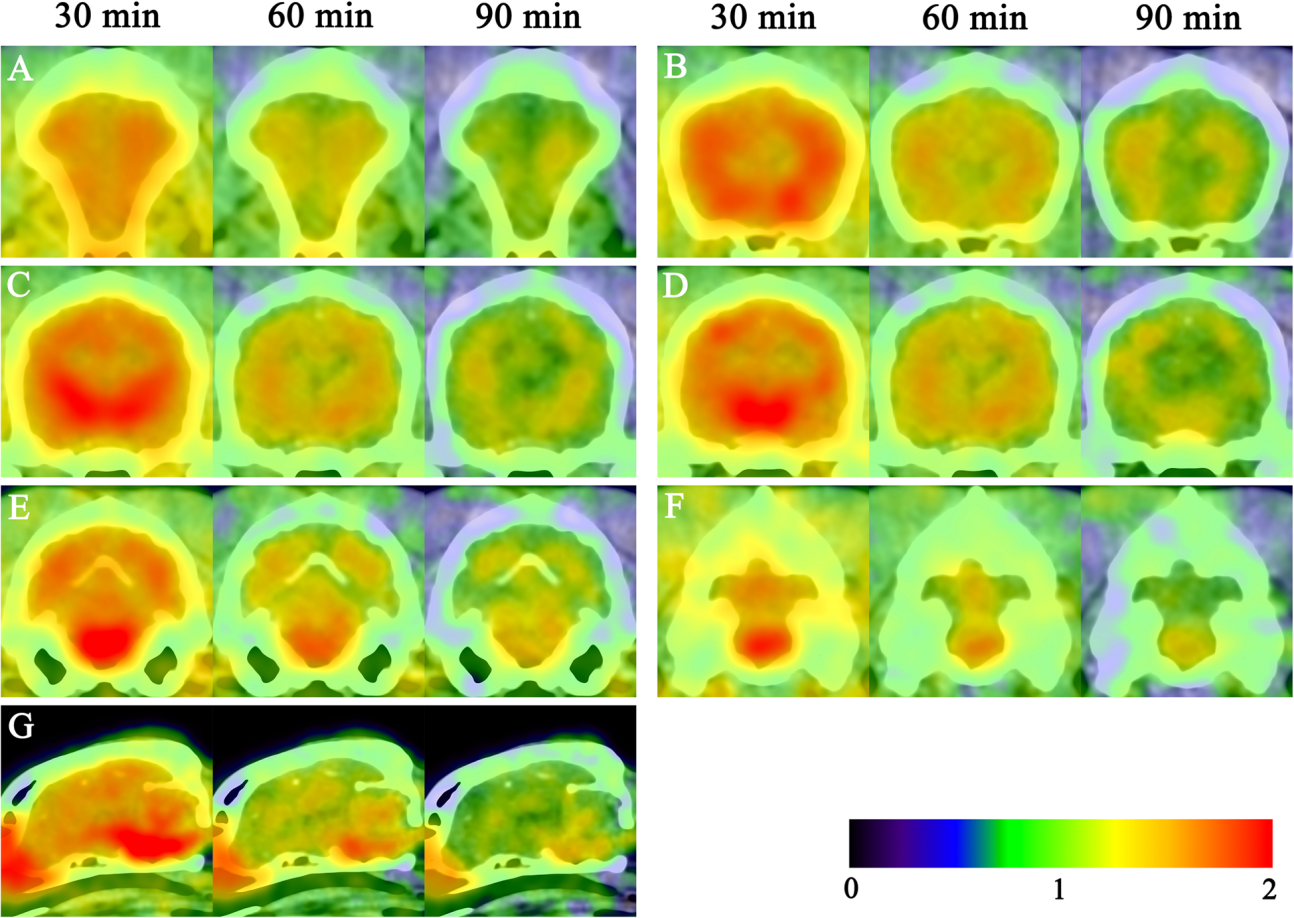
Static images of positron emission tomography (PET)/computed tomography (CT) fusion images at different times. Transverse and midsagittal ^18^F-flutemetamol brain PET images were taken at 30, 60, and 90 min post-injection of ^18^F-flutemetamol. The standardized uptake values (SUVs) for ^18^F-flutemetamol were displayed using a rainbow color scale. In PET/CT images, high accumulation of amyloid-beta protein is related to a reddish to yellowish color and low accumulation corresponds to a bluish to greenish color. The highest ^18^F-flutemetamol-uptake was observed in cerebral white matter and brainstem at all time points. The boundaries between the cerebral cortex and white matter became clear over time. **a** Frontal cortex, **b** anterior cingulate cortex, **c** parietal cortex, temporal cortex, and cerebral white matter, **d** posterior cingulate cortex, **e** occipital cortex, **f** cerebellar cortex, and **g** midbrain, pons, and medulla oblongata

All of the dogs were successfully scanned with the PET/computed tomography (CT) imaging procedure, and no adverse reactions were observed. Representative PET/CT fusion images are shown in Fig. 1. The standardized uptake values (SUVs) of the cerebral white matter and brainstem were always higher than those of the cerebral and cerebellar cortex. The boundaries between the cerebral cortex and white matter became clear over time.

The mean SUV values for each of the 11 brain regions as well as for the cortical region as a whole at different scanning times are listed in Table 1. The SUVs measured at 90 min post-injection were significantly lower than those measured at 30 min post-injection, in all brain regions (*P* = 0.0016). At 30 min post-injection, the highest mean SUV was observed in the pons. On the other hand, the lowest SUVs were observed in the occipital cortex and cerebellar cortex. ^18^F-flutemetamol uptake in the midbrain and pons was significantly higher than that in the occipital and cerebellar cortex (*P* < 0.02). At 60 min post-injection, the highest mean SUV was observed in the pons. On the other hand, the lowest SUV was observed in occipital cortex. ^18^F-flutemetamol uptake in the midbrain, pons, medulla oblongata, and cerebral white matter was significantly higher than that in the occipital cortex (*P* < 0.05). Additionally, the SUV of the pons was significantly higher than that of temporal and cerebellar cortex (*P* < 0.04). At 90 min post-injection, the highest mean SUV was observed in the pons. On the other hand, the lowest SUV was observed in cerebellar cortex. ^18^F-flutemetamol uptake in the pons and cerebral white matter was significantly higher than that in the occipital, temporal, and cerebellar cortex (*P* < 0.05). Among the cerebral cortices, the highest SUV was observed in the posterior cingulate cortex at 30 min, anterior cingulate cortex at 60 min, and frontal cortex at 90 min after tracer injection.

**Table 1:** Regional standardized uptake values (SUVs) for ^18^F-flutemetamol in normal canine brain regions at different scan times

	SUVs		
Region	30 min post-injection	60 min post-injection	90 min post-injection
Frontal cortex	0.65 ± 0.14	0.39 ± 0.09	0.29 ± 0.07*
Parietal cortex	0.62 ± 0.15	0.35 ± 0.08	0.26 ± 0.06*
Temporal cortex	0.63 ± 0.14	0.33 ± 0.08	0.22 ± 0.05*
Occipital cortex	0.53 ± 0.14	0.29 ± 0.07	0.22 ± 0.05*
Anterior cingulate cortex	0.70 ± 0.15	0.41 ± 0.09	0.28 ± 0.07*
Posterior cingulate cortex	0.71 ± 0.15	0.39 ± 0.09	0.28 ± 0.07*
Midbrain	1.25 ± 0.30^a,b^	0.65 ± 0.16^a^	0.42 ± 0.10*
Pons	1.35 ± 0.37^a,b^	0.80 ± 0.22^a,b,c^	0.53 ± 0.15*^,a,b,c^
Medulla oblongata	1.00 ± 0.24	0.64 ± 0.14^a^	0.44 ± 0.11*
Cerebral white matter	0.96 ± 0.23	0.68 ± 0.18^a^	0.51 ± 0.14*^,a,b,c^
Cerebellar cortex	0.53 ± 0.11	0.31 ± 0.08	0.21 ± 0.05*
Composite†	0.64 ± 0.14	0.36 ± 0.08	0.26 ± 0.06

Data were obtained at 30, 60, and 90 min post-tracer injection (*n* = 6). Values are the means ± SD. Superscripts * within rows indicate that the mean SUVs differ at 90 min compared with that at 0 min by *P* < 0.05; a, b, c within columns indicate that the mean SUVs differ from that of the occipital cortex, cerebellar cortex, and temporal cortex by *P* < 0.05; †Values for the composite comprise the average SUVs for the frontal, parietal, temporal, occipital, anterior cingulate, and posterior cingulate cortices; *SUV* standardized uptake value

### Regional SUVRs in the normal canine brain

Mean SUV ratio (SUVR) values for each of the 10 brain regions as well as whole cortical region at different scanning times are listed in Table 2. The SUVR measured at 90 min post-injection was significantly lower than that measured at 30 min post-injection in cerebral white matter (*P* = 0.282). At 30 min post-injection, the highest mean SUVR was observed in the pons. On the other hand, the lowest SUVR was observed in the occipital cortex. The relative ^18^F-flutemetamol uptake of the pons and midbrain, referenced to the cerebellar cortex, was significantly higher than that of the parietal, temporal, and occipital cortex (*P* < 0.02). Additionally, the SUVRs of the medulla oblongata and cerebral white matter were significantly higher than that of occipital cortex (*P* < 0.01). The SUVR of the pons was also significantly higher than that of the frontal cortex (*P* = 0.0338). At 60 min post-injection, the highest mean SUVR was observed in the pons. On the other hand, the lowest SUVR was observed in the occipital cortex. The relative ^18^F-flutemetamol uptake of the pons and cerebral white matter, referenced to the cerebellar cortex, were significantly higher than those of the temporal and occipital cortex (*P* < 0.05). Additionally, the SUVR of the pons was significantly higher than that of parietal cortex (*P* = 0.0249). The SUVRs of the midbrain and medulla oblongata were also significantly higher than that of occipital cortex (*P* < 0.02). At 90 min post-injection, the highest mean SUVR was observed in the pons. On the other hand, the lowest SUVR was observed in the occipital cortex. The relative ^18^F-flutemetamol uptake of the pons and cerebral white matter, referenced to the cerebellar cortex, was significantly higher than that of the temporal and occipital cortex (*P* < 0.01). Additionally, the SUVR of the medulla oblongata was significantly higher than that of occipital cortex (*P* = 0.0482). Among the cerebral cortices, the highest SUVR was observed in the posterior cingulate cortex at 30 min, anterior cingulate cortex at 60 min, and frontal cortex at 90 min after tracer injection.

**Table 2:** Regional standardized uptake value ratios (SUVRs) for ^18^F-flutemetamol in normal canine brain regions at different scan times

	SUVRs		
Region	30 min post-injection	60 min post-injection	90 min post-injection
Frontal cortex	1.23 ± 0.11	1.27 ± 0.30	1.38 ± 0.33
Parietal cortex	1.16 ± 0.12	1.15 ± 0.23	1.23 ± 0.25
Temporal cortex	1.18 ± 0.09	1.07 ± 0.20	1.06 ± 0.18
Occipital cortex	0.99 ± 0.06	0.95 ± 0.10	1.03 ± 0.14
Anterior cingulate cortex	1.33 ± 0.10	1.34 ± 0.27	1.32 ± 0.33
Posterior cingulate cortex	1.34 ± 0.10	1.28 ± 0.16	1.32 ± 0.28
Midbrain	2.35 ± 0.35^a,b,c^	2.13 ± 0.63^a^	2.03 ± 0.54
Pons	2.52 ± 0.48^a,b,c,d^	2.65 ± 0.91^a,b,c^	2.57 ± 0.89^a,b^
Medulla oblongata	1.88 ± 0.25^a^	2.10 ± 0.47^a^	2.12 ± 0.68^a^
Cerebral white matter	1.81 ± 0.24^a^	2.22 ± 0.65^a,b^	2.47 ± 0.72*^,a,b^
Composite†	1.25 ± 0.09	1.26 ± 0.20	1.27 ± 0.25

Values are SUVRs, using the cerebellar cortex as the reference region and are expressed as mean ± SD. Data were obtained at 30, 60, and 90 min post-tracer injection (*n* = 6). Superscripts * within rows indicate that the mean SUVR differs at 90 min as compared with that at 0 min by *P* < 0.05; a, b, c, d within columns indicate the mean SUVRs differ from that at the occipital cortex, temporal cortex, parietal cortex, and frontal cortex by *P* < 0.05; †Values for the composite comprise the average SUVRs for the frontal, parietal, temporal, occipital, anterior cingulate, and posterior cingulate cortices; *SUVR* Standardized uptake value ratio

### Time-activity curves for ^18^F-flutemetamol uptake

**Fig. 2:**
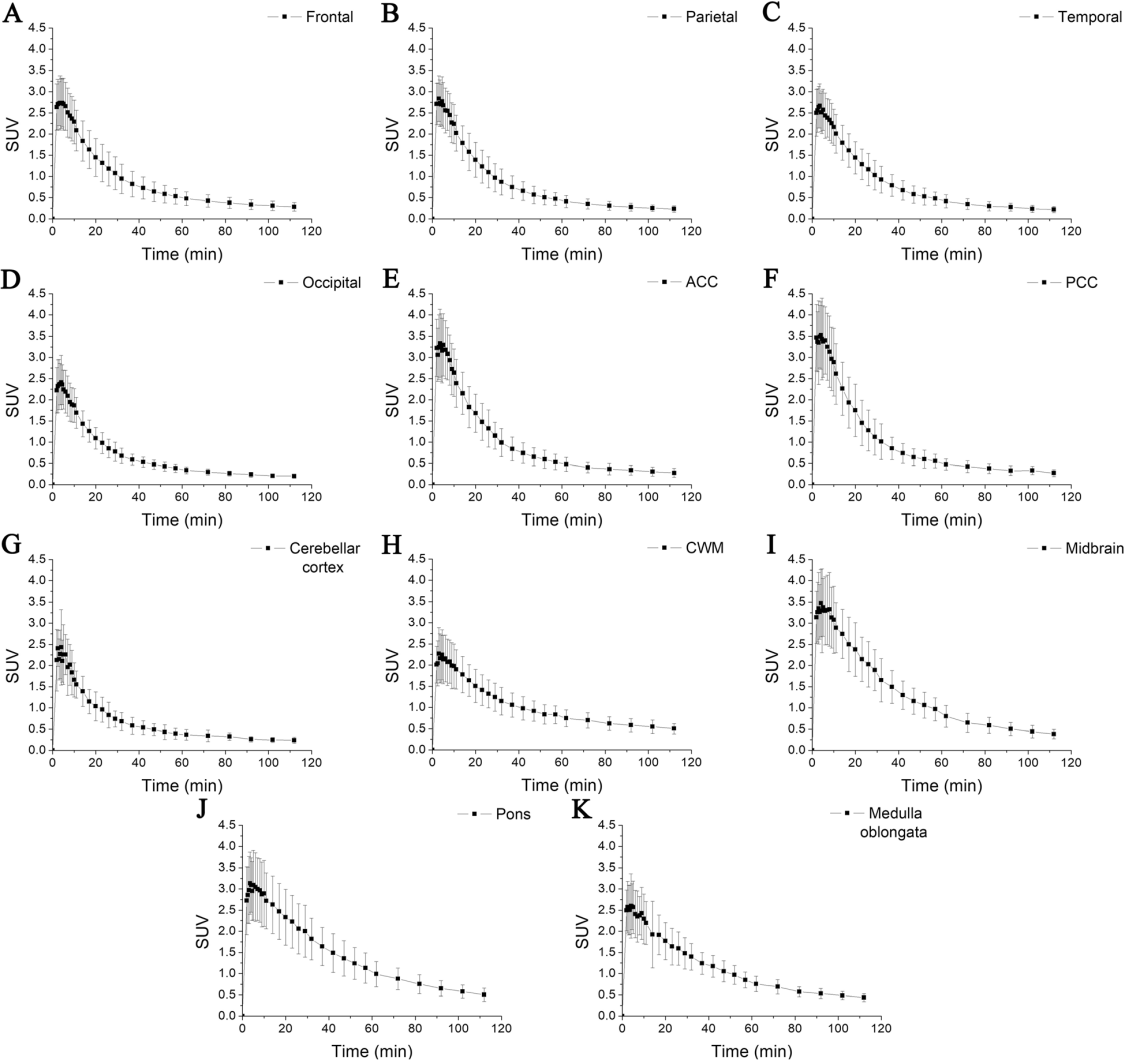
Time–activity curves for ^18^F-flutemetamol uptake over a 112-min period from the time of injection. Tracer uptake is expressed as means ± SD. The highest tracer uptake in all brain regions generally occurred between 3 and 4 min after injection. All brain regions showed similar time-activity curve patterns, reaching peak uptake early and showing rapid washout, except for the cerebral white matter. **a** Frontal cortex, **b** parietal cortex, **c** temporal cortex, **d** occipital cortex, **e** anterior cingulate cortex, **f** posterior cingulate cortex, **g** cerebellar cortex, **h** cerebral white matter, **i** midbrain, **j** pons, and **k** medulla oblongata

Time-activity curve analysis clearly showed different patterns of ^18^F-flutemetamol uptake for different regions within the 11 areas defined for evaluation in this study (Fig. 2). The highest tracer uptake in all brain regions generally occurred between 3 and 4 min after injection. All cerebral and cerebellar cortices showed similar time-activity curve patterns, reaching peaks (SUVs: 2.41–3.52) early (within 4 min) and showing rapid washout thereafter. Specifically, the peak SUVs of the frontal, parietal, temporal, occipital, anterior cingulate, posterior cingulate, and cerebellar cortices were 2.73 ± 0.64, 2.84 ± 0.54, 2.66 ± 0.52, 2.41 ± 0.64, 3.33 ± 0.80, 3.52 ± 0.79, and 2.43 ± 0.89 at 3.5, 3, 3.5, 4, 3.5, 4, and 4 min post-injection, respectively. On the other hand, the cerebral white matter showed a lower peak uptake (2.27 ± 0.62) at 3 min post-injection and much slower washout over a period of 109 min after the peak time (Fig. 2h). The SUV of the cerebral white matter started to exceed those of all cerebral cortices after 29 min post-injection. The uptake patterns of ^18^F-flutemetamol within the brainstem were also similar to those of cerebral cortices. The peak SUVs of the midbrain, pons, and medulla oblongata were 3.47 ± 0.80, 3.13 ± 0.73, and 2.60 ± 0.76 at 4, 3.5, and 4 min post-injection, respectively. However, the uptake of the midbrain, pons, and medulla oblongata exceeded those of all cerebral cortices markedly earlier than did the cerebral white matter SUV, at 7, 10, and 20 min after tracer injection, respectively.

## Discussion

The precise pathogenesis of AD is still unclear but probably known to be associated with accumulation of Aβ and neurofibrillary tangles [4]. For this reason, animal models for pathology of AD are needed to find the mechanisms of Aβ deposits. Aged dogs naturally show cognitive dysfunctions that share several characteristics of AD, such as amyloid deposits in the cerebral cortex and clinical signs [22–24]. Dogs are animal which is easy to control and share many things (including environment and diet) with human. Dogs also provide pharmacokinetic model when converting results to human clinical trials, because they are not the same but very similar to human [30]. Therefore, the application of dogs as animal models for AD provides theories of human brain aging and therapeutic options. To use as an animal model for AD and set diagnostic criteria for CDS, this study using ^18^F-flutemetamol PET/CT was performed. Among 6 amyloid PET tracers, ^18^F-flutemetamol was chosen because it is the most used and the only tracer available commercially in Korea when this study was performed.

In the present study, in addition to using SUV, which is commonly obtained in ^18^F-fluorodeoxyglucose-PET, we introduced the use of SUVR. Recently, SUVR has been used as a representative index of regional brain uptake of amyloid imaging agents. Abundant deposit of Aβ protein also occurs in the brains of cognitively and behaviorally normal elderly people, who lack noticeable neurological dysfunction [31]. For this reason, as in previous human amyloid PET imaging studies, we chose the cerebellar cortex as the primary reference region, because this region is reportedly remarkably free of pathologic fibrillar plaques [32].

Clinical PET have been performed using static acquisition in human. Therefore, the static mode was studied to identify normal data for clinical application. Similar to human studies [7], the highest ^18^F-flutemetamol uptakes among substructures of the cerebral cortex were observed in the frontal and cingulate cortex. Quantification of regional tracer uptake can help clinicians to interpret Aβ PET images, as a complement to visual evaluation. The SUVRs calculated for canine brain regions in this study were very similar to a report in which brain PET scans were performed to assess ^18^F-flutemetamol uptake in a variety of brain regions in healthy humans [7]. The mean human SUVRs of the frontal, parietal, temporal, occipital, anterior cingulate, and posterior cingulate cortices were 1.34 ± 0.33, 1.18 ± 0.32, 1.22 ± 0.26, 1.35 ± 0.19, 1.38 ± 0.34, and 1.40 ± 0.36, respectively. These values compared favorably to the 90 min SUVRs reported for normal dogs in the current study. The composite cortical SUVR of ^18^F-flutemetamol in the normal canine brain (1.27 ± 0.25) was also similar to that of other amyloid tracers, such as ^11^C-Pittsburg compound B (1.38 ± 0.32) and ^18^F-florbetaben (1.32 ± 0.19), in healthy humans [8].

The dynamic mode was carried out to identify temporal uptake of ^18^F-flutemetamol by brain regions. Uncharged thioflavin-T derivates, the raw material for producing ^18^F-flutemetamol, are known to have good entry into and clearance from the brain, with a high affinity for Aβ plaques [27]. In terms of the temporal distribution of ^18^F-flutemetamol, many studies have reported that amyloid-detecting tracer uptake in the brain peaked within a few min and decreased rapidly thereafter [9, 33, 34]. In the present study, the patterns observed were similar to those of ^18^F-flutemetamol [9] and ^11^C-Pittsburg compound B [33] in healthy humans. However, the time at which the SUVs of the frontal cortex and cerebral white matter crossed each other differed between healthy humans (about 35 min post-injection) and normal dogs (about 16 min post-injection). Potential reasons for differences in this crossing time between species include differences in the clearance rate and the amount of physiological amyloid deposits. There is also difference of this crossing time between healthy and AD people [9]. Compared to healthy people (about 35 min post-injection), the time at which the SUVs of the frontal cortex and cerebral white matter cross each other in AD people was about 80 min post-injection [9]. Furthermore, after this cross time, while the gap of uptake level between cerebral cortex and cerebral white matter in healthy people is relatively large, that in AD people is relatively small [9]. Although, more information is needed to set the acquisition time of ^18^F-flutemetamol PET in dogs, considering these differences in addition to further study on temporal distribution in CDS dogs, it might be helpful to set the acquisition time of ^18^F-flutemetamol PET in dogs.

Imaging with PET in AD patients have provided an important tool for evaluation of the etiology and diagnosis [5–13, 32, 34, 35]. In ^18^F-fluorodeoxyglucose-PET, decreased glucose metabolism was identified in posterior cingulate, temporoparietal, and prefrontal association cortex, showing correlation with dementia severity [35]. In ^18^F-flutemetamol PET in AD patient, the SUVR of cerebral cortex was known to be significantly greater than in older healthy people (1.76 ± 0.23 vs 1.30 ± 0.26) [7]. Although decreased glucose metabolism and increased amyloid deposits, identified by PET, were not used as definitive diagnostic criteria, PET imaging have been used to increase the objectivity of an Alzheimer’s diagnosis.

The main limitation of this study was that the data were obtained only from normal young beagle dogs. Therefore, further studies need to be conducted in other breeds of dog to be generalization beyond beagle dogs. Furthermore, to identify accurate differences between normal and CDS dog and establish diagnostic criteria for CDS, it will be important to perform further studies in dogs with CDS. It would be also necessary to have different age groups with normal brain investigated in order to draw conclusions about the physiological aging process of the canine brain. Another limitation was that dynamic mode was performed after static mode with a week interval. The pharmacokinetics of ^18^F-flutemetamol in dogs have not been conducted, so nothing is known yet, but the ^18^F-flutemetamol that left a week ago by static acquisition may have influenced the results of dynamic acquisition.

## Conclusion

To our knowledge, the use of ^18^F-flutemetamol PET/CT in clinically normal dogs has not been reported previously. Uptake of ^18^F-flutemetamol can readily be quantified using the SUVR, taking the cerebellar cortex as a reference region. The results obtained for the normal canine brain were similar to those reported in human studies. These data provide the baseline for further investigations and diagnosis of CDS in veterinary medicine. Additionally, ^18^F-flutemetamol PET/CT may be a valuable method for understanding the role of Aβ in non-CDS amyloidopathies, given that amyloid PET imaging of conditions other than CDS is an area of active research.

## Methods

### Animals

Six beagle dogs (two males and four females; DooYeol biotech, Seoul, Republic of Korea), weighing 8.72 ± 1.05 [mean ± standard deviation (SD)] kg and ranging in age from 3 to 4 years (mean ± SD, 3.19 ± 0.24 years) were used in this study. All dogs were healthy without a history of neurological disorder; they had no abnormal signs in physical and neurological examinations. They were also tested for metabolic diseases by establishing a complete blood count and serum chemistry profile. The dogs were acclimated for more than 2 weeks and housed under the following conditions: an ambient temperature of 20 ± 2 °C, relative humidity of 50 ± 10%, air ventilation rate of 10 cycles per h, and a 12:12 h light:dark cycle. The dogs were fed a commercial dry food (L.I.D. Limited Ingredient Diets® Potato & Duck Dry Dog Formula, Natural balance, SanFrancisco, CA, USA) twice a day, and fresh water was supplied continuously throughout the experimental period. This experimental protocol was approved by the Institutional Animal Care and Use Committee (CBNUA-1168-18-01) of the Laboratory Animal Research Center of Chungbuk National University. After the experiment, all dogs were adopted as companion animals.

### Animal preparation and anesthesia for PET/CT scanning

All dogs were fasted for at least 12 h before the induction of anesthesia, but had free access to drinking water. After intravenous (IV) catheter placement at the saphenous vein, anesthesia was induced with propofol (6–8 mg/kg IV; Provive, Myungmoon Pharm, Seoul, Republic of Korea). Endotracheal intubation was performed, and anesthesia was maintained with isoflurane (Terrell; Piramal Critical Care, Bethlehem, PA, USA) at 2.5 to 3% of the inspired volume during scanning, in a circle rebreathing system. Intermittent positive pressure ventilation was applied and tidal volume for ventilation was 10–20 mL/kg body weight with a respiratory frequency of 10–15 breaths per minute. A urethral catheter was inserted under sterile conditions, which included wearing sterile gloves. Dogs received normal saline (0.9% NaCl) solution (5 mL/kg/h) during anesthesia. They were positioned in sternal recumbency within the PET/CT gantry. Vital signs, such as heart rate, oxygen saturation (SPO_2_), end-tidal CO_2_-concentration, and blood pressure were continuously monitored, and the dogs were warmed with a warming pad (Equator; SurgiVet, Saint Paul, MN, USA).

### ^18^F-flutemetamol PET/CT scanning

The PET/CT system used in this study was a Discovery-STE (General Electric Medical Systems, Waukesha, WI, USA). An 8-slice helical CT scanner and a cylindrical PET device with 13,440 bismuth germanium oxide crystals arranged in 24 rings were included in the Discovery-STE system. The crystals, which have a dimension of 4.7 × 6.3 × 30 mm^3^, are organized in blocks of 8 × 6, coupled to a single photomultiplier with four anodes. The PET system provides 47 images at 3.27-mm intervals covering an axial field-of-view of 256 mm.

The injection dose (3.083 MBq/kg) of ^18^F-flutemetamol (Vizamyl, GE Healthcare, Arlington Heights, IL, USA) was calculated based on the dosing scheme of ^18^F-fluorodeoxyglucose in pediatric patient published by the 2010 North American Consensus Guidelines [36].

For static emission acquisition, ^18^F-flutemetamol was injected IV into a saphenous vein as a slow bolus, followed by flushing with 5 ml of 0.9% normal saline. A low-dose CT scan was performed prior to each PET scan. Twenty-minute PET images were acquired three times, from 30 to 50 min, from 60 to 80, and from 90 to 110 min after tracer injection.

For dynamic emission acquisition, images were obtained 1 week after obtaining static acquisitions. A transmission scan of 1-min duration was acquired first in the two-dimensional mode. Subsequently, a low-dose CT scan was performed and ^18^F-flutemetamol (3.083 MBq/kg) was administered intravenously. Dynamic three-dimensional PET images were acquired for 120 min starting at 2 min after the ^18^F-flutemetamol injection. A total of 31 frames were acquired at 6 × 30 s, 6 × 60 s, 7 × 180 s, 6 × 300 s, and 6 × 600 s.

The images were reconstructed with iterative techniques (four iterations with 70 subsets) with a slice thickness of 3.27 mm, matrix size of 128 × 128, with pixel sizes of 2 mm for the emission scan. Corrections for attenuation and scatter were applied. A Gaussian post-reconstructing smoothing filter with a 5-mm full-width at half-maximum was used to achieve uniform image resolution across sites.

### Image analysis

**Fig. 3:**
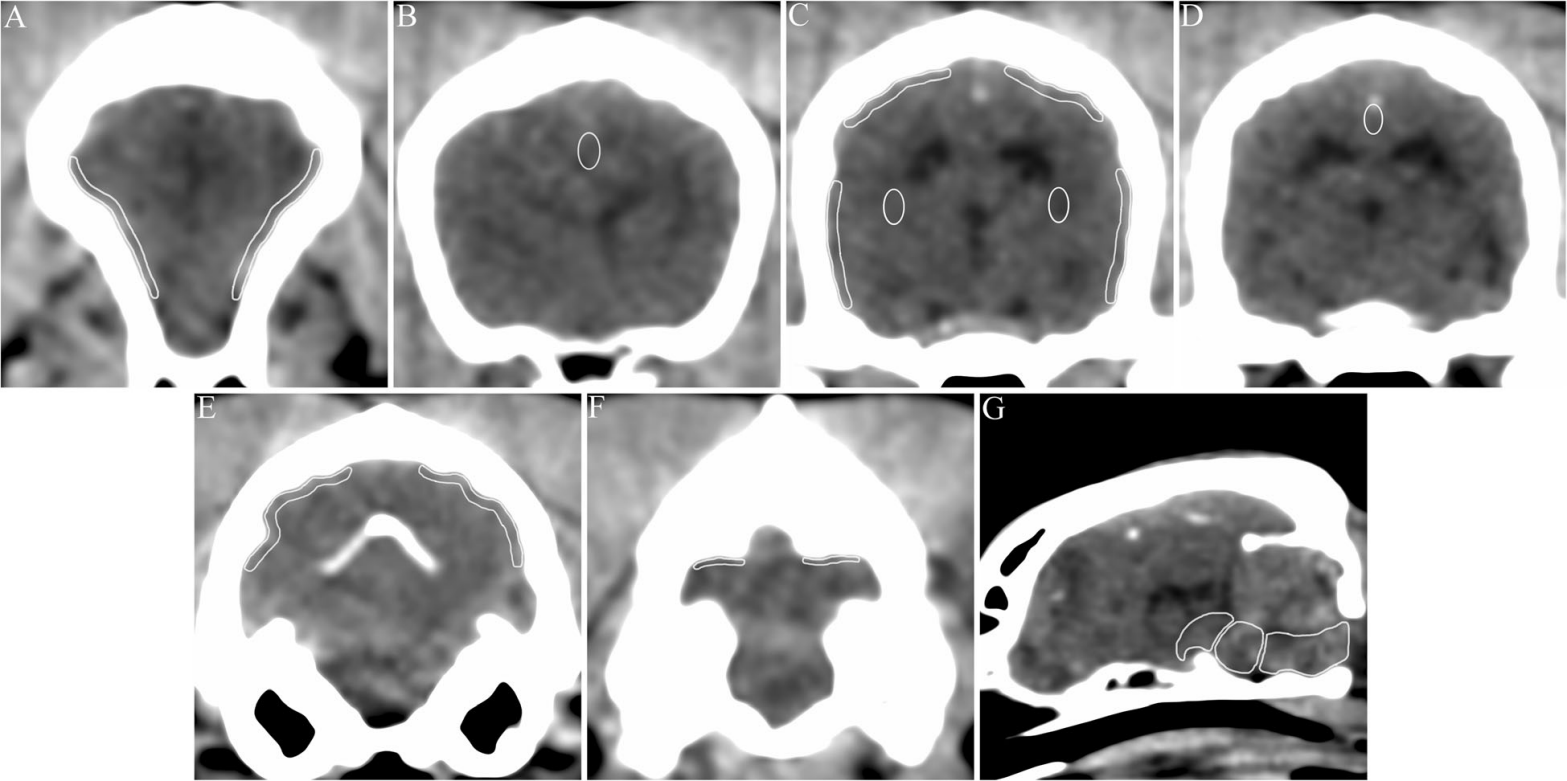
Schematic representative images of regions of interest (ROIs) defined on computed tomography (CT) images. White lines are enhanced examples of ROIs assessed in this study. **a** Frontal cortex, **b** anterior cingulate cortex, **c** parietal cortex, temporal cortex, and cerebral white matter, **d** posterior cingulate cortex, **e** occipital cortex, **f** cerebellar cortex, **g** midbrain, pons, and medulla oblongata

OsiriX MD v10.0 (Pixmeo Sarl, Geneva, Switzerland) was used to analyze PET images. The regions of interest (ROIs) for each brain area were drawn manually on transverse (frontal cortex, parietal cortex, temporal cortex, occipital cortex, anterior cingulate cortex, posterior cingulate cortex, cerebellar cortex, and cerebral white matter) and midsagittal (midbrain, pons, and medulla oblongata) CT images (Fig. 3). For semiquantitative image analysis, ROIs were drawn over three consecutive slices except for the cerebellum (two slices) and brainstem (one slice). All PET analyses were performed using the same standardized ROI in the brain. The ROIs were then transferred to the corresponding region of PET/CT fusion images, and the SUV (average tissue concentration of ^18^F-flutemetamol [kBq/ml] / total injected dose [kBq] / body weight [g]) was calculated for each ROI. The SUVRs were also calculated for each ROI, to quantify brain uptake of the tracer. Dividing the SUVs of the individual target areas by that of the reference region (cerebellar cortex) yielded regional SUVRs. In addition to the regional SUVs and SUVRs, composite SUVs and SUVRs were obtained by calculating the mean SUV and SUVR from the frontal, temporal, parietal, occipital, anterior cingulate, and posterior cingulate cortices. Time–activity curves were generated over the entire acquisition period to determine the temporal course of regional brain ^18^F-flutemetamol uptake.

### Statistical analysis

Data were analyzed using GraphPad Prism 6 software (GraphPad Software Inc., San Diego, CA, USA). The Friedman test was employed to test data within one group for changes over time, and, where overall significance was found, Dunn’s multiple-comparison post-hoc test was used to determine the origin of differences. Differences between groups were assessed using the Kruskal–Wallis test. If differences were significant, Dunn’s test was used for comparisons between groups. All values in each table were expressed as means ± SD. Differences were considered significant at *P* < 0.05.

## Data Availability

The datasets used and/or analyzed during the current study are available from the corresponding author on reasonable request.

## Abbreviations

AD: Alzheimer’s disease
Aβ: Amyloid-beta
CDS: Cognitive dysfunction syndrome
CT: Computed tomography
MRI: Magnetic resonance imaging
PET: Positron emission tomography
ROI: Region of interest
SD: Standard deviation
SUV: Standardized uptake value
SUVR: Standardized uptake value ratio
